# Cascading effects of composts and cover crops on soil chemistry, bacterial communities and the survival of foodborne pathogens

**DOI:** 10.1111/jam.15054

**Published:** 2021-04-06

**Authors:** N. Devarajan, J.A. McGarvey, K. Scow, M.S. Jones, S. Lee, S. Samaddar, R. Schmidt, T.D. Tran, D.S. Karp

**Affiliations:** ^1^ Department of Wildlife, Fish, and Conservation Biology University of California, Davis Davis CA USA; ^2^ Agricultural Research Service U.S. Department of Agriculture Albany CA USA; ^3^ Department of Land, Air and Water Resources University of California, Davis Davis CA USA; ^4^ Department of Entomology Washington State University Pullman WA USA; ^5^ Tree Fruit Research and Extension Center Washington State University Wenatchee WA USA

**Keywords:** biological soil amendment of animal origin, co‐management, compost, food safety, healthy soil, Listeria, manure, organic, Salmonella, soil amendment

## Abstract

**Aims:**

Recent foodborne disease outbreaks have caused farmers to re‐evaluate their practices. In particular, concern that soil amendments could introduce foodborne pathogens onto farms and promote their survival in soils has led farmers to reduce or eliminate the application of animal‐based composts. However, organic amendments (such as composts and cover crops) could bolster food safety by increasing soil microbial diversity and activity, which can act as competitors or antagonists and reduce pathogen survival.

**Methods and Results:**

Leveraging a study of a 27‐year experiment comparing organic and conventional soil management, we evaluate the impacts of composted poultry litter and cover crops on soil chemistry, bacterial communities and survival of *Salmonella enterica* and *Listeria monocytogenes*. We found that bacterial community composition strongly affected pathogen survival in soils. Specifically, organic soils managed with cover crops and composts hosted more macronutrients and bacterial communities that were better able to suppress *Salmonella* and *Listeria*. For example, after incubating soils for 10 days at 20°C, soils without composts retained fourfold to fivefold more *Salmonella* compared to compost‐amended soils. However, treatment effects dissipated as bacterial communities converged over the growing season.

**Conclusions:**

Our results suggest that composts and cover crops may be used to build healthy soils without increasing foodborne pathogen survival.

**Significance and Impact of the Study:**

Our work suggests that animal‐based composts do not promote pathogen survival and may even promote bacterial communities that suppress pathogens. Critically, proper composting techniques are known to reduce pathogen populations in biological soil amendments of animal origin, which can reduce the risks of introducing pathogens to farm fields in soil amendments. Thus, animal‐based composts and cover crops may be a safe alternative to conventional fertilizers, both because of the known benefits of composts for soil health and because it may be possible to apply amendments in such a way that food‐safety risks are mitigated rather than exacerbated.

## Introduction

Foodborne disease outbreaks associated with the consumption of fresh produce have emerged as a major public health concern (World Health Organization 2015). In the United States, 24 814 illnesses and 88 deaths were attributed to fresh produce from 2000 to 2015 (CDC 2020), with the majority of cases (>80%) attributed to three pathogens: *Listeria monocytogenes*, *Salmonella enterica* and Shiga toxin‐producing *Escherichia coli* (STEC) (Dewey‐Mattia *et al*. 2018).

In response to new food‐safety regulations and pressure from their buyers, fresh produce farmers across the United States altered their farming practices to mitigate risks associated with foodborne pathogens (Baur *et al*. 2016).

While foodborne pathogens can enter and contaminate farms through a variety of pathways, animal‐based soil amendments represent particular cause for concern (Park *et al*. 2012). Many studies document a higher prevalence of foodborne pathogens in fields amended with raw animal manures relative to conventional fertilizers (Islam *et al*. 2004; Mukherjee *et al*. 2007; Marti *et al*. 2013; Gu *et al*. 2018). Moreover, foodborne pathogen growth and survival may be limited by nutrients in soils (Ongeng *et al*. 2015); thus, applying animal‐based composts to the soil could enhance foodborne pathogen survival by providing them with key nutrient resources (Shah *et al*. 2019). That said, the risk of introducing pathogens onto farms can be mitigated through proper composting techniques (Islam *et al*. 2004; FDA 2017; Gu *et al*. 2018). Still, citing concerns about introducing foodborne pathogens and promoting their survival in soils, many produce farmers have stopped applying both raw manures and animal‐based composts in their farming operations (Baur *et al*. 2016; Khalsa and Brown 2017; Olimpi *et al*. 2019). For example, in a surveillance study, fewer than a quarter of California fruit and nut producers (*n* = 306) reported using fully composted soil amendments and less than 10% used non‐composted soil manure (Baur *et al*. 2016).

At the same time, farmers recognize the benefits of applying composts for soil health (Khalsa and Brown 2017). Indeed, the addition of organic matter to soil via organic amendments (e.g. composts, animal manure, mulches and green waste) improves soil quality as well as key biotic and abiotic properties (Ros *et al*. 2006; Chaudhry *et al*. 2012; Chen *et al*. 2017; Tautges *et al*. 2019; Kranz *et al*. 2020). For example, green waste from cover crops can increase soil organic matter, prevent nutrient leaching and increase nitrogen content (Fernandez *et al*. 2016; Reed‐Jones *et al*. 2016; Vukicevich *et al*. 2016; Tautges *et al*. 2019). Similarly, composts can increase soil nutrient concentrations (Zhong *et al*. 2009; Wichuk *et al*. 2011; Wei *et al*. 2017), nutrient holding capacity (Clark *et al*. 1998) and soil organic matter (Bulluck *et al*. 2002; Jones *et al*. 2019). By adding nutrients to soils (especially organic carbon), composts also enhance microbial biomass, activity and diversity (Hartmann *et al*. 2015; Lupatini *et al*. 2016; Jones *et al*. 2019). Finally, by altering soil characteristics and microbial diversity, composts can help suppress crop pests and pathogens (Weller *et al*. 2002; Brown and Tworkoski 2004; Larkin 2015; De Corato 2020).

Compost may also suppress human foodborne pathogen survival by increasing indigenous microbial diversity and biomass (Sidhu *et al*. 2001; Henault‐Ethier *et al*. 2016; Jones *et al*. 2019). Soil microbes compete for space and resources (e.g. carbon, nitrogen and other nutrients) with foodborne pathogens, and thus may prevent pathogenic bacteria from persisting (Henault‐Ethier *et al*. 2016). As a result, higher soil microbial diversity is often associated with lower foodborne pathogen survival in soils (Locatelli *et al*. 2013; Erickson *et al*. 2014; Williams *et al*. 2015; Jones *et al*. 2019; Baker *et al*. 2020).

Still, while the direct risks associated with introducing pathogens into the farm environment are well understood, the impacts of animal‐based composts and other soil amendments on foodborne pathogen survival in soils are less clear. Long‐term agricultural experiments offer unique opportunities to trace the effects of farm management on soil properties, microbial communities and foodborne pathogen suppression. Here, we leverage a 27‐year farming system field experiment to understand the cascading effects of soil management on human foodborne pathogen suppression in soils. Specifically, we measured, in laboratory microcosms, how composted poultry litter and/or cover crops affect the ability of agricultural soils to suppress the foodborne pathogens *L. monocytogenes* and *S. enterica* (*Listeria* and *Salmonella*, hereafter).

We organized our study around three questions. First, how does long‐term soil management with cover cropping and compost applications affect soil physicochemical properties and bacterial communities? Second, do soil physicochemical properties and bacterial communities influence the ability of *Salmonella* and *Listeria* to persist in soils? Finally, how does long‐term soil management, namely compost additions and cover cropping, affect foodborne pathogen survival in soils?

## Materials and methods

### Experimental design

Our study was conducted at the Century Experiment at the Russell Ranch Sustainable Agriculture Facility, University of California, Davis, USA. Since 1993, 72 64 × 64 m (1 acre) fields have been continuously managed under nine replicated cropping systems, arranged in a randomized complete block design with 2‐year rotations. Our experiment focused on 12 fields managed under a corn–tomato rotation, all growing corn during 2019 (when our experiments occurred). Corn was planted in two rows per bed, and drip irrigated throughout the growing season.

We compared four soil management treatments, each replicated across three fields, which we refer to as (i) organic fields: managed with composted poultry litter and winter cover crops but no synthetic fertilizers, (ii) conventional fields: managed with synthetic fertilizers but no compost or cover crops, (iii) cover‐crop only fields: mixed fields managed with cover crops and fertilizers but no composts and (iv) compost only fields: mixed fields managed with composts and fertilizers but no cover crops. Unlike the other treatments that were established in 1993, the latter treatment (compost only fields) was recently converted from a conventional wheat–tomato system in 2018.

For fields receiving composts, composted poultry litter was broadcast across the field and incorporated into the soil during the fall at an average rate of 4 t per ha. Composts were not tested for foodborne pathogens prior to application. For fields receiving cover crops, a winter cover crop mixture of 90 kg ha^−1^ *Vicia faba* (bell bean), 22·5 kg ha^−1^ *Vicia villosa* (hairy vetch) and 28 kg ha^−1^ *Avena sativa* (oats) was planted in the fall. In the following spring, winter cover crops were mowed and incorporated into the soil with disking. For fields receiving synthetic fertilizers, 56 kg N ha^−1^ of 8‐24‐6 (N‐P‐K) starter fertilizer was applied in early April. Treatment plots that received synthetic fertilizers were fertilized with ammonium sulphate at a total rate of 180 kg N ha^−1^. Fertilization rates were designed to hold macronutrient inputs constant across the four treatments.

### Soil sampling

Two soil types occur at Russell Ranch: Yolo silt loam (fine‐silty, mixed non‐acid, thermic Typic Xerorthents) and Rincon silty clay loam (fine, montmorillonitic, thermic Mollic Haploxeralfs) (Wolf *et al*. 2018). We collected soils from experimental plots on four occasions: before corn was planted (17 April 2019), 1 month after planting (1 July 2019), 2 months after planting (6 August 2019) and 2‐days before harvest (17 September 2019). On each sampling day, a hand auger (8 cm width) was used to collect soil (0–15 cm depth) from 10 locations, randomly distributed across each 1‐acre plot. The 10 samples were composited into a sterile 1‐l plastic bag and transported to the laboratory in a cooler with cool packs.

All soil samples were sieved, using an 8‐mm sieve and initial soil moisture content was measured by drying at 55–60°C for 24 h in a Salvis oven (Cole‐Parmer, Chicago, IL). Homogenized samples were analysed for (i) macronutrients including nitrate (NO_3_‐N; chromotropic acid method), ammonia (NH_4_‐N; salicylate method), phosphorous (P; Olsen method), potassium (K; ammonium acetate method), calcium (Ca; ammonium acetate method) and magnesium (Mg; ammonium acetate method); (ii) micronutrients including sodium (Na; ammonium acetate method), zinc (Zn), iron (Fe), manganese (Mn), copper (Cu) and boron (B; DTPA—sorbitol extraction method for all micronutrients except sodium), soil texture (i.e. % sand, silt and clay; hydrometer method), pH (SMP soil buffer pH) and organic matter (Walkley‐Black titration method) by Soiltest Farm Consultants (Moses Lake, WA) (Gavlak *et al*. 2003).

### Soil DNA extraction, amplicon sequencing and analysis

DNA was extracted from 0·25 g of soil from each sample using the DNeasy PowerSoil Pro Kit (Qiagen, Hilden, Germany), following the manufacturer’s protocol. DNA concentrations were quantified, stored at −20°C and shipped to Integrated Microbiome Resource (IMR), Halifax, Canada for library preparation and sequencing. Bacterial 16S rRNA gene fragments (V4‐V5 region) were amplified using universal primers (515F: GTGYCAGCMGCCGCGGTAA; 926R: CCGYCAATTYMTTTRAGTTT (Walters *et al*. 2016). Amplicons were sequenced on a MiSeq platform, with 2 × 250 bp configuration. For 16S rDNA data analyses, reads were first trimmed to remove primer sequences using Cutadapt (Martin 2011). All further processing was performed using DADA2 (ver. 1.12) (Callahan *et al*. 2016) in R (R Core Team 2020). In DADA2, the forward and reverse reads were truncated to 250 and 200 bp, respectively, based on quality profile plots along with maxEE = c(2,2) as an additional parameter. Following trimming and quality processing, reads were dereplicated and unique reads were denoised, considering the error rates calculated with the ‘learnErrors’ function. Next, paired sequences were merged, and chimera were removed. SILVA database release 132 was used to assign taxonomy to the 16S reads (Quast *et al*. 2013). Reads assigned to chloroplasts, mitochondria, Archaea and Eukarya were removed.

A total of 12 140 unique bacterial amplicon sequence variants (ASVs) were obtained. To ensure that rare ASVs did not influence trends, the ASV table obtained was further filtered using the package ‘OTUtable’ (Linz *et al*. 2017) in R to limit ASV’s to contain <0·1% and <0·01% of the total reads within a sample. Alpha diversity (i.e. Shannon and Simpson diversity) was calculated at the ASV level using ‘phyloseq’ (McMurdie and Holmes 2013). Uneven sequencing depths were normalized using a variance stabilizing transformation method with the DESeq2 package (Love *et al*. 2014). Beta‐diversity (Bray–Curtis, Euclidian and Unifrac dissimilarities) was calculated with phyloseq. Raw reads were archived in Sequences Read Archives (SRA) of National Center for Biotechnology Information (NCBI) with accession PRJNA656855.

### Pathogen strains

*Listeria monocytogenes* strain RM15994NR is a spontaneous nalidixic acid (nal) and rifampicin (rif)‐resistant mutant of *L. monocytogenes* strain RM15994, an isolate from the 2011 multistate cantaloupe‐associated *Listeria* outbreak (CDC 2011). *Listeria monocytogenes* RM15994NR was generated by growing *L. monocytogenes* strain RM15994 in 1 ml of tryptic soy broth (TSB) (Remel Inc., San Diego, CA) at 37°C for 24 h shaking (200 rev min^−1^). Fifty microliters of this culture were transferred to 1 ml of TSB+1·5 mg l^−1^ nal (Fisher Bioreagents, Pittsburgh, PA) and incubated an additional 24 h as above. The culture was passed through TSB + 3·0 and then 6·0 mg l^−1^ nal as above and 100 µl of the resulting culture was plated onto modified Oxford agar (Oxoid, Basingstoke, UK)+50 mg l^−1^ nal (MOX + nal). An isolate from this plate was inoculated into 1 ml of TSB + 1·5 mg l^−1^ rif (Fisher Bioreagents) and again into TSB + 3·0 mg l^−1^ rif, and finally into TSB + 6·0 mg l^−1^ rif and plated onto MOX + nal+100 mg l^−1^ rif (MOX+nal+rif) and incubated for 48 h at 37°C. *Salmonella enterica* strain RM3363NR is a spontaneous nal rif‐resistant mutant generated from *S. enterica* strain RM3363, an isolate from the 2002 multistate cantaloupe‐associated *Salmonella* outbreak (Anderson *et al*. 2002). *Salmonella enterica* strain RM3363 was generated using the same protocol as above except that the final cultures were plated onto McConkey agar base (Remel Inc., San Diego, CA)+40 mmol l^−1^ maltose+nal+rif. Resistant bacteria were stored in TSB+nal+rif+20% glycerol at −80°C until needed.

### Pathogen suppression experiments

We chose to conduct a pathogen‐suppression bioassay in laboratory microcosm, rather than field conditions, both because it was not feasible to introduce pathogens into the growing environment and because the laboratory afforded better control over ambient conditions. All bioassays were performed on soil samples within 24 h of collection in a bio‐safety level 2 laboratory (USDA, CA). *L. monocytogenes* RM15994NR and *S. enterica* RM3363NR were grown for 24 h in TSB at 37°C shaking (200 rev min^−1^). Fifty grams of soil were added to sterile specimen cups (Fisher scientific, Pleasanton, CA) and inoculated with 10–10 µl drops of the 24 h *L. monocytogenes* culture or 10–10 µl drops of a 10‐fold dilution in phosphate‐buffered saline (1× PBS) of the 24 h *S. enterica* culture by randomly placing the drops around the surface of the soil (average bacterial inoculum concentration: *L. monocytogenes* ~9·55E + 07 and *S. enterica* ~7·67E + 07 CFU per 50 g of soil). Cups were covered and incubated at 20°C for 10 or 30 days and enumerated for the number of the pathogens remaining. Specifically, 50 g of each of the contaminated soil samples was added to a sterile blender jar and blended with 50 ml of PBS. Ten‐fold serial dilutions of the homogenized soil samples were performed using PBS and 100 µl was plated onto modified MOX+nal+rif+ 40 mg l^−1^ cycloheximide (Oxoid) agar plates or MacConkey agar base+40 mmol l^−1^ maltose+nal+rif+ 40 mg l^−1^ cycloheximide and quantified for *L. monocytogenes* or *S. enterica*, respectively. Plates were incubated at 37°C for 24 h (*S. enterica*) or 48 h (*L. monocytogenes*) and the number of colonies counted. The mean population of pathogens per ml solution was converted to mean per gram population in soil (wet weight) and averaged among triplicates.

### Statistical analysis

We implemented linear mixed models (LMMs) to explore relationships between management treatments, physicochemical properties, bacterial communities and pathogen survival. All models included a random effect of ‘field’ to account for multiple visits to the same field. Fixed effects varied by analysis.

We first modelled the effects of soil management on soil physicochemical properties. We used principal components analysis (PCA) to derive indices of macronutrients (i.e. nitrate, ammonia, phosphorous, potassium, sulphur, calcium and magnesium), micronutrients (i.e. zinc, iron, manganese, copper, boron and sodium) and soil texture (i.e. sand, silt and clay). The first principal component of each analysis corresponded to increases in macronutrients, micronutrients and percent sand and explained 97%, 74% and 96% of the variation, respectively. Along with these three principal components, we also modelled effects of soil management on soil pH, initial moisture content and organic matter. Fixed effects included (i) a binary variable indicating whether compost was applied, (ii) a binary variable indicating the presence of cover crops and (iii) the number of elapsed days since the beginning of the experiment (to account for changes over the growing season). We also included interactions between compost and cover crop treatments to account for synergistic effects in the fully organic or conventional treatments. Finally, we included interactions between compost treatments and elapsed days, as well as cover crop treatments and elapsed days, to account for changes in treatment effects over the growing season.

We modelled the effects of soil management on soil bacterial diversity (Simpson and Shannon diversity) using the same model specification as above. We then used permutational multivariate analysis of variance (permanova) to determine whether bacterial communities were distinct between different treatments and different sampling dates (using each beta‐diversity metric). For post‐hoc analysis, we used permanova to compare communities between pairs of treatments. Finally, to determine whether differences in community composition between treatments shifted over time, we calculated the multivariate distance from each ‘compost’ sample to the centroid of all samples that did not receive compost (Anderson *et al*. 2006; Karp *et al*. 2018). Centroid distances were separately calculated for each survey date. We then modelled centroid distances as a function of the number of elapsed days since the first survey. Distances were log transformed to satisfy normality assumptions. For all analyses of bacterial diversity and composition, we created separate models to predict variation in metrics using all the ASVs in each sample (unfiltered) as well as excluding ASVs that constituted <0·1% and <0·01% of the total reads within a sample to ensure models were robust to rare ASVs.

Next, we modelled the effects of soil physicochemical properties on bacterial diversity. Due to collinearity between variables (variance inflation factors >3; Zuur *et al*. 2009), we conducted two separate analyses; one for macronutrients, micronutrients, texture, and initial moisture content and another for organic matter and pH. Analyses were repeated for each measure of soil diversity and ASV filtering procedure. To assess effects on community composition, we used permanova, again separating organic matter and pH from the other fixed effects.

Finally, we assessed the effects of soil physicochemical properties, bacterial diversity, bacterial community composition and soil management treatments on pathogen suppression. Pathogen suppression was measured as the fraction of pathogens remaining after 10 and 30 days of incubation in soils (i.e. final mean probable number (MPN) divided by the MPN of the initial inoculum). Variables were transformed to meet normality assumptions. For *Salmonella*, we calculated the fraction remaining and then implemented a log transformation (adding 1 to the inoculum and the final measured MPN). For *Listeria*, we calculated the fraction remaining and then implemented a quarter‐root transformation.

We assessed the effects of soil physicochemical properties on pathogen suppression using the same model specification as analyses of physicochemical properties on soil bacterial diversity. To assess the effects of bacterial diversity, we implemented separate models for each diversity predictor and filtering procedure. To assess the effects of bacterial composition, we used Bray–Curtis, Euclidian and Unifrac dissimilarities to perform non‐metric multidimensional scaling (NMDS) and then extracted the first two NMDS axes of each analysis. These axes were then used as predictors of pathogen suppression. Finally, to assess the effects of soil management treatments on pathogen suppression, we used nearly the same model specification as in prior analyses of soil bacterial diversity and physicochemical properties. However, because pathogen suppression rates seemed to exhibit nonlinear seasonal dynamics, we also included higher‐order elapsed day terms (squared and cubed terms).

We always ensured data conformed to model assumptions (i.e. heteroscedasticity and normality), transforming data when normality was not initially achieved. We then assessed the importance of fixed effects in two ways to ensure our results were robust. First, we implemented a model averaging procedure, using the MuMIn package in R to obtain the non‐shrinkage variance estimates for each fixed effect (i.e. averaging only over models that included the focal fixed effect (Barton *et al*. 2020; R Core Team 2020). Second, we implemented backwards model selection, comparing nested models with likelihood ratio tests (LRTs) and iteratively eliminating fixed effects until removing any additional effects would reduce model performance (i.e. LRT *P* < 0·05). When interactions explained significant variation, main effects were retained in the final models used to generate figures.

## Results

**Figure 1:**
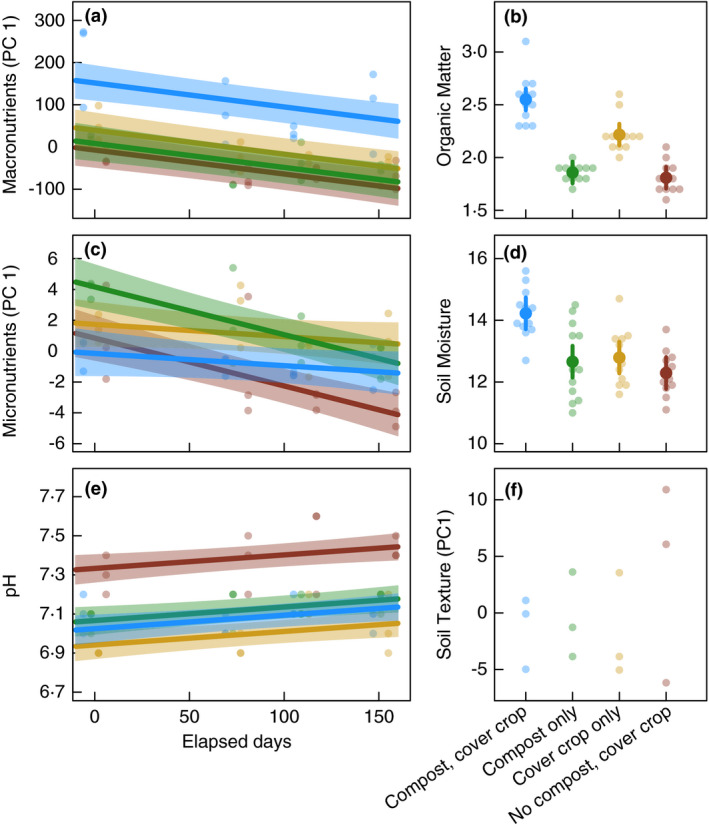
Long‐term soil management alters soil chemical properties. Macronutrient (principal component axis 1; Panel a), organic matter (Panel b) and initial moisture content (Panel d) levels peaked in organic soils (i.e. soils with composts and cover crops). Conventional soils (i.e. soils with synthetic fertilizers only) were more nutrient poor and had higher pH levels (Panel e). Micronutrients (principal component axis 1; Panel c) exhibited complex dynamics, peaking at the beginning of the growing season in compost‐only soils and reaching their lowest levels at harvest in conventional soils. Soils did not differ in texture (principal component axis 1; Panel f). Points represent individual soil samples, coloured according to each soil management treatments. For soil properties that exhibited significant changes over the growing season (Panels a/c/e), lines represent predictions from linear mixed models and shaded regions are 95% confidence intervals. For other soil properties (Panels b/d/f), large solid points and vertical lines represent predicted treatment means and 95% confidence intervals, respectively. Treatment groups: 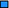 (organic = cover crop + compost), 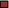 (conventional), 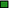 (compost‐only) and 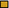 (cover‐crop only).

We found that soil management altered soil chemical properties, with cascading implications for bacterial communities and foodborne pathogen survival. Organic soils (i.e. soils with composts and cover crops but no fertilizers) had higher levels of soil macronutrients, organic matter and initial moisture content (Fig. 1a,b,d; Table S1) and reduced soil pH (Fig. 1e). Effects of soil management on micronutrients were more complex. At the beginning of the growing season, soils in the compost‐only treatment (i.e. soils with composts and fertilizers but no cover crops) had the most micronutrients (Fig. 1c). However, micronutrients declined over time in the compost‐only and conventional treatments. As a result, conventional soils (i.e. soils with fertilizers but no composts or cover crops) had fewer micronutrients than the other treatments by harvest. As expected, soil management did not affect soil texture, as in Tautges *et al*. 2019 (Fig. 1f).

**Figure 2:**
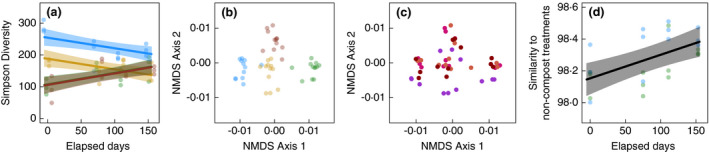
Bacterial communities differ between soil management treatments, especially at the beginning of the growing season. (Panel a) Bacterial diversity (Simpson index) peaked in soils with compost and cover crops; however, differences between treatments attenuated over the growing season. Points are jittered slightly along the *x*‐axis for visualization purposes. (Panel b) Each of the four management treatments hosted a distinct bacterial community. (Panel c) Community composition also shifted over the growing season. (Panel d) Specifically, as the growing season progressed, bacterial communities became increasingly similar between compost and non‐compost treatments. Here, similarity is measured as the inverse, multivariate distance from each of the compost samples to the centroid of all non‐compost samples (both conventional and cover‐crop only treatments; see methods). Points are individual soil samples, coloured according to soil management treatments (Panels a/b/d) or the elapsed days since the beginning of the experiment (Panel c). In panels a/d, lines correspond to predictions from linear mixed models; shaded regions are 95% confidence intervals. In NMDS plots (panels b/c), distances between points correspond to differences in bacterial community composition. Treatment groups: 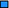 (organic = cover crop + compost), 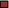 (conventional), 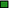 (compost‐only) and 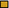 (cover‐crop only) (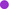 0 days; 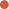 111 days; 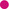 75 days; 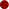 153 days).

Soil management also affected bacterial communities. Together, cover crops and composts tended to increase bacterial diversity (Fig. 2a; Table S2); however, the 2‐year‐old compost‐only treatment could not be differentiated from the conventional treatment. Each soil management treatment hosted a distinct community (Fig. 2b; Table S3). Still, treatment differences attenuated over the growing season (Fig. 2c,d; Tables S3 and S4), both in terms of bacterial diversity and composition. Soil chemical properties may have underlined the effects of soil management on bacterial communities. Soils with more macronutrients, more organic matter, higher initial moisture content and fewer micronutrients tended to host more diverse bacterial communities (Fig. S1; Table S5). Similarly, community composition strongly shifted with changes in macronutrients, organic matter, pH, and, to a lesser extent, with changes in micronutrients and soil texture (based on *F* statistics and *P* values; Fig. S2; Table S6). Initial soil moisture content did not affect community composition. All effects were robust to ASV filtering procedures (see methods).

**Figure 3:**
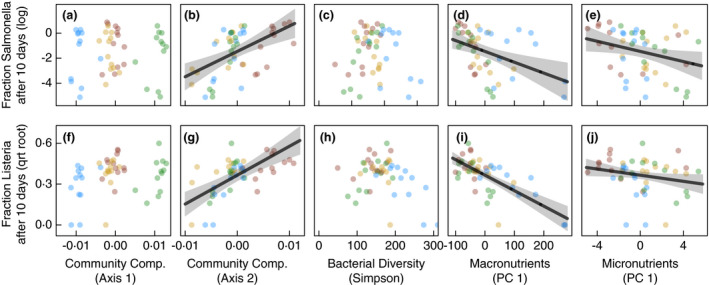
Bacterial communities and soil nutrient content are associated with foodborne pathogen suppression. Pathogen suppression is measured as the mean probable number (MPN) of *Salmonella* or *Listeria* after 10 days of incubation, divided by the initial MPN of the inoculant (followed by log and quarter‐root transformations for *Salmonella* and *Listeria*, respectively; see methods). *Salmonella* and *Listeria* concentrations exhibited steeper declines in soils with more macronutrients (Panel d/i), more micronutrients (Panel e/j) and bacterial communities (Panel b/g) more similar to those present in soils with composts and/or cover crops (i.e. NMDS axis 2 in Fig. 2b). The first NMDS axis explaining variation in bacterial community composition (Panel a/f) and bacterial diversity (Simpson index; Panel C/H) did not affect foodborne pathogen suppression. Points represent soil samples, coloured by management treatment. Lines represent predictions from linear mixed models; shaded regions are 95% confidence intervals. Treatment groups: 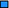 (organic = cover crop + compost), 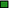 (conventional), 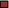 (compost‐only) and 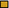 (cover‐crop only).

**Figure 4:**
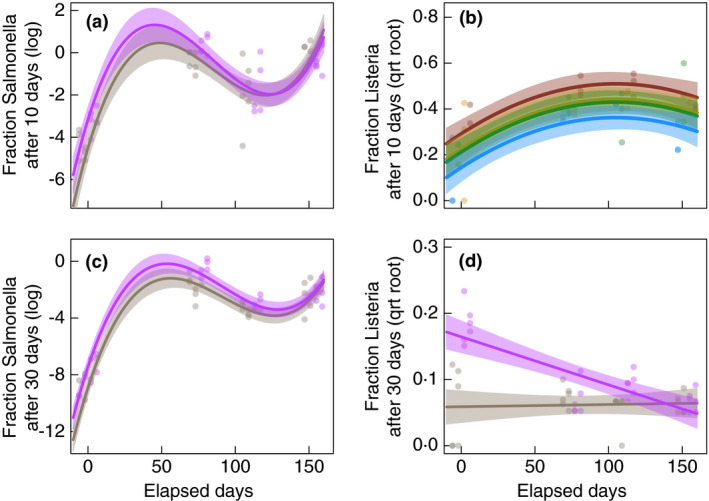
Composted soils are more pathogen suppressive than soils without composts, but only at the beginning of the growing season. Pathogen suppression is measured as the fraction of *Salmonella* or *Listeria* remaining in soil samples after 10 or 30 days of incubation (see methods). At the beginning of the growing season, both compost treatments (compost/cover crop as well as compost‐only) exhibited greater reductions in *Salmonella* following 10 (Panel a) and 30 days (Panel c) of incubation than both treatments without compost (cover‐crop only and no compost/cover crop). However, *Salmonella* suppression fluctuated over the growing season, with treatment differences converging at harvest. Results were similar for 30‐day *Listeria* incubations: both compost treatments resulted in greater suppression than both non‐compost treatments at the beginning but not at the end of the growing season. In contrast, *Listeria* reductions after 10‐day incubations were greatest in the organic treatment (compost/cover crop), intermediate in the cover‐crop only and compost‐only treatments and lowest in conventional soils (no compost/cover crop), regardless of sampling date. Points are individual soil samples, with the four sampling dates jittered slightly for visualization. In panels a/c/d, points and lines are coloured to reflect significant differences between both compost treatments *vs* both non‐compost treatments. In panel b each treatment is coloured differently to reflect significant differences between them. Lines represent predictions from linear mixed models; shaded regions are 95% confidence intervals. Treatment groups: 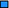 (organic = cover crop+compost), 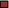 (conventional), 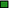 (compost‐only) and 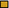 (cover‐crop only) (a,c,d: 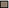 both treatments with compost; 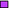 both treatments without compost).

Soil physicochemical properties and bacterial communities were associated with foodborne pathogen suppression. Across all samples, *Salmonella* was reduced to ~62% of the initial inoculation levels after 10 days and ~15% after 30 days. Nonetheless, *Salmonella* survival exhibited marked fluctuations over the growing season. Specifically, survival was very low before corn was planted but increased after plants had established. Across the season, soils with more macronutrients and micronutrients were more likely to suppress *Salmonella* following both 10‐ and 30‐day incubations (Fig. 3 and Fig. S3; Table S7). Other soil properties (i.e. pH, organic matter, initial moisture content, texture) and bacterial diversity did not affect *Salmonella* suppression (Fig. 3 and Figs S3–S5; Tables S7 and S8). Bacterial community composition, however, strongly affected *Salmonella* suppression after both 10‐ and 30‐day incubations (Fig. 3; Table S8). Models predicted *Salmonella* concentrations would either grow by ~75% after 10 days of incubation or decline to ~4% of its initial levels, depending on the bacterial community composition. Specifically, *Salmonella* persisted longer in soils with bacterial communities more similar to those present in conventional soil treatments (high values of NMDS axis 2 in Fig. 2b). This effect was robust across community composition metrics and ASV filtration procedures. Finally, we observed greater *Salmonella* reductions in both compost treatments compared to both treatments that did not receive compost at the beginning of the growing season (Fig. 4; Table S9); however, all treatments ultimately converged to yield similar levels of *Salmonella* suppression by harvest. Cover crops alone did not influence *Salmonella* survival.

*Listeria* levels attenuated more rapidly than *Salmonella* (average of 3 and 0·02% remaining after 10‐ and 30‐day incubations, respectively). After 10 days, we found that *Listeria* exhibited steeper declines in soils with more macronutrients, more micronutrients, more organic matter and less sand (Fig. 3 and Fig. S4; Table S7). While initial soil moisture content, pH and soil microbial diversity were not significant predictors, *Listeria* also persisted best in soils with bacterial communities more similar to those present in the conventional treatment (Fig. 3; Table S8). As a result, models predicted that the least amount of *Listeria* persisted after 10‐day incubations in organic soils and the most persisted in conventional soils (Fig. 3; Table S9). Compost‐only and cover‐crop only treatments exhibited intermediate suppression. Perhaps because *Listeria* levels were always very low, soil physicochemical properties and bacterial community composition did not influence the amount of *Listeria* remaining after 30‐day incubations (Figs S3 and S5; Tables S7 and S8). However, *Listeria* did exhibit steeper declines over 30‐day incubation periods in more diverse soils and in compost‐amended soils, at the beginning but not at the end of the growing season (Fig. 3 and Fig. S3; Tables S8 and S9). Cover crops alone did not influence *Listeria* survival after 30 days.

## Discussion

**Figure 5:**
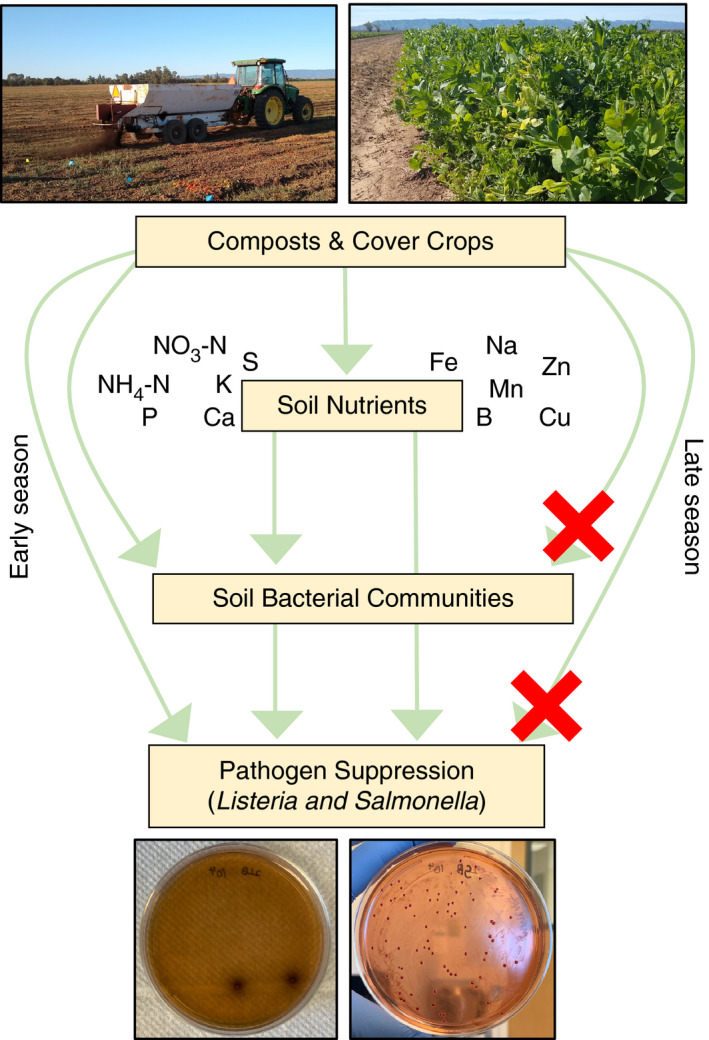
Results synthesis. Our study suggests that composts and cover crops increase soil nutrients, driving a shift in bacterial communities and an increase in some measures of bacterial diversity. These cascading effects on soil chemical properties and bacterial communities enhance the ability of organic soils to suppress foodborne pathogens. Importantly, however, as bacterial communities increasingly converge between soil management treatments over the growing season, so too does the ability of soils to suppress foodborne pathogens. As a result, soils experiencing very different management histories are largely equivalent in their ability to suppress *Salmonella* and *Listeria* at harvest.

Our results demonstrate that long‐term soil management alters soil chemical properties, with cascading implications for bacterial communities and foodborne pathogen survival (Fig. 5). Specifically, we found that the introduction of cover crops and composts as soil amendments increased soil macronutrients, organic matter and soil moisture, in turn causing marked shifts in bacterial communities. Perhaps because soils’ abilities to suppress pathogens are correlated with their biotic and abiotic properties (van Elsas *et al*. 2012; Williams *et al*. 2015), *Listeria* and *Salmonella* concentrations declined more rapidly in soils that were amended with composts. However, differences between treatments dissipated over the growing season as bacterial communities converged. Finally, we found that including cover crops in a conventional rotation, but without compost, had no impact on pathogen survival.

That composts and cover crops increased soil nutrients, organic matter, and bacterial diversity is consistent with prior long‐term soil management studies (Zhong *et al*. 2009; Wichuk *et al*. 2011; Wei *et al*. 2017). Through adding carbon and building organic matter, composts help create large pools of stored nutrients, thereby maintaining soil fertility over the long term (Clark *et al*. 1998; Bulluck *et al*. 2002; Liu *et al*. 2007; Kranz *et al*. 2020). Creating organic‐rich soils with few synthetic compounds in turn provides heterogeneous habitat niches which can be occupied by diverse microbial communities (Lupatini *et al*. 2016). As a result, higher microbial diversity is often documented in soils amended with organic inputs (managed with cover crops and/or composts) compared to conventional soils (receiving only crop residues as organic inputs) (Chaudhry *et al*. 2012; Chen *et al*. 2017; Wang *et al*. 2018). Congruently, we found that bacterial diversity increased with organic matter and macronutrients, reaching its highest levels in soils treated with both cover crops and composts. Interestingly, however, bacterial diversity did not differ between conventional soils and soils that only received composts (i.e. no cover crops), perhaps because the compost‐only treatment was established recently (whereas all other treatments have been managed for 27 years). Still, bacterial communities strongly differed in composition between every treatment, even the recently established compost‐only treatment.

Differences in bacterial diversity and composition between treatments were greatest prior to corn planting but dissipated over the growing season. Bacterial community composition and functional properties often fluctuate seasonally, responding to seasonal changes in plant phenology, solar radiation, moisture, temperature and nutrient levels (van Diepeningen *et al*. 2006; Postma *et al*. 2008; Toljander et al. 2008; Orr *et al*. 2012; Koranda *et al*. 2013; Ishaq *et al*. 2020). For example, one recent study found that seasonal fluctuations in water content and nutrient availability (i.e. nitrate concentrations) drove marked shifts in bacterial composition over the corn growing season (Romero‐Salas *et al*. 2021). In our study, macronutrient concentrations declined over the growing season and bacterial communities responded strongly to these changes. Thus, one possibility is that, as the growing season progressed, composts and/or cover crops broke down and caused the differences between treatments to dissipate. Another possibility is that corn plants homogenized soil conditions across treatments as they grew, causing bacterial communities to converge. Indeed, as plants establish, grow, take up nutrients and release organic compounds, soil chemistry shifts and microbial communities are sensitive to these changes (Macdonald *et al*. 2004; Paterson *et al*. 2007).

Changes in soil characteristics and bacterial community composition between treatments and across the growing season strongly correlated with *Salmonella* and *Listeria* survival in soils. For example, after 10‐day incubations, our models predicted 2% of the *Salmonella* would remain in soils rich in macronutrients *vs* 55% in soils with few macronutrients. These findings suggest that although foodborne pathogens can be resource limited in soils (Ongeng *et al*. 2015), increasing the low nutrient content of agricultural soils may ultimately create a less hospitable environment for foodborne pathogens by fuelling indigenous bacterial communities (Williams *et al*. 2015). We also found that both pathogens persisted better in soils with bacterial communities associated with conventional management (i.e. no compost or cover crops). Correspondingly, at the beginning of the season, models predicted that 4x–5x more *Salmonella* persisted in soils without compost relative to soils with compost (10‐day incubation: 4% *vs* 1% remaining; 30‐day incubation: 0·05% *vs* 0·01% remaining). However, as bacterial communities converged between treatments over the growing season, so did *Salmonella* persistence. Surprisingly, we found no evidence that *Salmonella* survival differed between the organic (i.e. cover crops and composts) and compost‐only treatment, even though the compost‐only treatment was recently established. Moreover, cover crops alone did not reduce *Salmonella* persistence.

Effects of soil management on *Listeria* were broadly similar but differed in three key ways. First, following 10‐day incubations, *Listeria* survival was lowest in organic soils, intermediate in compost‐only and cover‐crop only soils, and highest in conventional soils. Second, effects persisted throughout the entire growing season for 10‐day (but not 30‐day) incubations. Finally, after 30‐day incubations, *Listeria* suppression was associated with bacterial diversity rather than community composition. Across all treatments, we found that pathogen survival was much lower at the beginning of the growing season, suggesting that compost amendment timing, seasonal changes in soil characteristics, produce growth stages and soil bacterial communities may be key to pathogen persistence.

Together, these results suggest that adding composts to soils does not promote foodborne pathogen survival and may even lead to soils that are more suppressive of *Salmonella* and *Listeria*. However, before suggesting that soil amendments could be used as a strategy to improve food safety, at least four important caveats must be noted. First, our study suggested strong temporal dynamics, with treatment effects dissipating over the growing season. While making soils more pathogen suppressive at any time could improve food safety, developing practices that continue to function through harvest would be ideal. Second, our study was conducted in laboratory conditions with soil from experimental farms. A key next step would be inoculating soils with pathogens in the field to assess survival in ambient conditions (Williams *et al*. 2015). Third, we used a 27‐year soil manipulation experiment to assess cover crop and compost impacts on pathogen survival. Shorter experiments are necessary to determine how long it would take for farmers to make their soils more pathogen suppressive. Nonetheless, because *Salmonella* survival was indistinguishable between the 27‐year organic treatment (compost and cover crops) and the more recent compost‐only treatment, it is possible that shorter‐term compost additions could also make soils more pathogen suppressive. Finally, prior studies have documented positive, negative and neutral effects of compost additions on pathogen persistence depending on the compost type, the pathogen, prior soil management history and other factors (Erickson *et al*. ; Vivant *et al*. 2013; Shah *et al*. 2019; Sharma *et al*. 2019). Though less often studied, cover crop effects on foodborne pathogens may also be context‐dependent (Reed‐Jones *et al*. 2016) and warrant further studies to assess their short‐term and long‐term impacts on enteric pathogens.

Because animal‐based composts may introduce foodborne pathogens onto farms (Park *et al*. 2012), many fresh‐produce farmers feel tension between promoting soil health and food safety (Baur *et al*. 2016; Khalsa and Brown 2017; Olimpi *et al*. 2019). Soil microbial communities may help reconcile this perceived trade‐off. Specifically, we found that application of composted poultry litter and cover crops increased soil macronutrients and enhanced bacterial communities that were better able to suppress *Salmonella* and *Listeria*. As a result, both pathogens exhibited lower survival in composted soils at the beginning of the growing season. Though the degree to which these findings can be generalized to commercial field conditions and other crop production systems remains to be seen, our findings suggest that compost amendments do not promote the survival of *Salmonella* and *Listeria*. Instead, by altering soil bacterial communities, it is possible that compost actually reduces pathogen survival, as has been previously shown with plant pathogens (Weller *et al*. 2002). Instituting proper composting techniques (e.g. heat treatments) and waiting periods before harvest can substantially reduce the risk of introducing pathogens onto farms (Gurtler *et al*. 2018). Abandoning animal‐based composts should thus be reconsidered, both because of the known benefits of composts for soil health and because it may be possible to apply amendments in such a way that food‐safety risks are mitigated rather than exacerbated.

## Conflict of Interest

The authors declare that they have no conflict of interest.

## Authors’ contributions

N.D., J.A.M, K.S., M.J., R.S. and D.S.K. designed the research; N.D., S.L. and T.T. collected the data; N.D., S.S. and D.S.K. analysed the data; N.D. and D.S.K. led the writing of the manuscript; all authors contributed critically to subsequent drafts.

## Supporting information


**Figure S1**. Effects of soil physicochemical properties on soil bacterial diversity.
**Figure S2**. Effects of soil physicochemical properties on bacterial community composition.
**Figure S3**. Effects of soil physicochemical properties and bacterial communities on pathogen suppression.
**Figure S4**. Effects of soil physicochemical properties on pathogen suppression after 10 days of incubation.
**Figure S5**. Effects of soil physicochemical properties on pathogen suppression after 30 days of incubation.
**Table S1**. Effects of soil management on soil bacterial diversity.
**Table S2**. Effects of soil management on soil bacterial diversity.
**Table S3**. Effects of soil management on bacterial community composition.
**Table S4**. Changes in the dissimilarity between bacterial communities present in compost *vs* non‐compost treatments over the growing season (measured as multivariate distances from each ‘compost sample’ to the centroid of all non‐compost samples; see methods).
**Table S5**. Effects of soil physicochemical properties on soil bacterial diversity.
**Table S6**. Effects of soil physicochemical properties on bacterial community composition.
**Table S7**. Effects of soil physicochemical properties on pathogen suppression (fraction of *Salmonella* or *Listeria* remaining after 10‐ and 30‐day incubations).
**Table S8**. Effects of soil bacterial diversity and community composition on pathogen suppression (fraction of *Salmonella* or *Listeria* remaining after 10‐ and 30‐day incubations).
**Table S9**. Effects of soil management on pathogen suppression (fraction of *Salmonella* or *Listeria* remaining after 10‐ and 30‐day incubations).
