# Supplementary material for: Cascading effects of composts and cover crops on soil chemistry, bacterial communities and the survival of foodborne pathogens
Source: J Appl Microbiol. 2021 Apr 6;131(4):1564–77. doi: 10.1111/jam.15054 (PMC8519115; doi:10.1111/jam.15054)
Supplement: Supplementary file 1 — Figure S1. Effects of soil physicochemical properties on soil bacterial diversity. Figure S2. Effects of soil physicochemical properties on bacterial community composition. Figure S3. Effects of soil physicochemical properties and bacterial communities on pathogen suppression. Figure S4. Effects of soil physicochemical properties on pathogen suppression after 10 days of incubation. Figure S5. Effects of soil physicochemical properties on pathogen suppression after 30 days of incubation. Table S1. Effects of soil management on soil bacterial diversity. Table S2. Effects of soil management on soil bacterial diversity. Table S3. Effects of soil management on bacterial community composition. Table S4. Changes in the dissimilarity between bacterial communities present in compost vs non‐compost treatments over the growing season (measured as multivariate distances from each ‘compost sample’ to the centroid of all non‐compost samples; see methods). Table S5. Effects of soil physicochemical properties on soil bacterial diversity. Table S6. Effects of soil physicochemical properties on bacterial community composition. Table S7. Effects of soil physicochemical properties on pathogen suppression (fraction of Salmonella or Listeria remaining after 10‐ and 30‐day incubations). Table S8. Effects of soil bacterial diversity and community composition on pathogen suppression (fraction of Salmonella or Listeria remaining after 10‐ and 30‐day incubations). Table S9. Effects of soil management on pathogen suppression (fraction of Salmonella or Listeria remaining after 10‐ and 30‐day incubations). [file JAM-131-1564-s001.pdf]

Cascading effects of composts and cover crops on soil chemistry, bacterial communities, and the survival of foodborne pathogens

Supplementary Tables and Figures

Table S1: Effects of soil management on soil bacterial diversity. Table includes coefficients (b), test statistics (Z), and P-values from model averaging as well as test statistics (χ^2) and P-values from likelihood-ratio tests, comparing models with and without each predictor. Models with significant interactions always retained constituent fixed effects.

	Macronutrients (PC 1)			Micronutrients (PC 1)			Moisture Content		
	β	Model Averaged Z, P	Model Selection χ^2, P	β	Model Averaged Z, P	Model Selection χ^2, P	β	Model Averaged Z, P	Model Selection χ^2, P
Compost	33.20	0.82, 0.41	Included	3.25	3.09, <0.01	Included	0.56	1.10, 0.027	Included
Cover crop	75.80	1.73, 0.08	Included	1.00	0.71, 0.48	Included	0.70	1.38, 0.17	Included
Elapsed days	-4.17	2.17, 0.03	21.0, <0.01	-0.30	4.22, <0.01	Included	-0.02	0.64, 0.52	1.10, 0.29
Compost : Cover crop	96.40	2.23, 0.03	5.6, 0.02	-5.21	3.87, <0.01	13.1 <0.01	1.07	1.69, 0.09	4.12, 0.04
Compost : Elapsed days	-2.66	1.18, 0.24	1.75, 0.19	0.03	0.34, 0.73	0.15, 0.70	-0.02	0.40, 0.69	0.20, 0.65
Cover crop : Elapsed days	-3.80	1.70, 0.09	3.34, 0.07	0.23	2.64, 0.01	7.72, <0.01	-0.02	0.43, 0.67	0.23, 0.63
	**Organic Matter**			**pH**			**Soil Physical Properties (PC1)**		
	β	Model Averaged Z, P	Model Selection χ^2, P	β	Model Averaged Z, P	Model Selection χ^2, P	β	Model Averaged Z, P	Model Selection χ^2, P
Compost	0.09	0.78, 0.43	Included	-0.23	3.38, <0.01	Included	-1.82	0.51, 0.61	0.44, 0.51
Cover crop	0.45	3.93, <0.01	Included	-0.39	7.04, <0.01	Included	-3.10	0.90, 0.37	1.20, 0.55
Elapsed days	-0.01	0.94, 0.35	3.81, 0.05	0.01	2.28, 0.02	7.37, 0.01	Only sampled once		
Compost : Cover crop	0.28	2.21, 0.03	5.89, 0.02	0.35	5.07, <0.01	17.8, <0.01	4.56	0.60, 0.55	0.73, 0.39
Compost : Elapsed days	-0.01	0.92, 0.36	1.04, 0.31	-0.01	1.57, 0.12	2.87, 0.09	Only sampled once		
Cover crop : Elapsed days	-0.01	0.97, 0.33	1.13, 0.29	0.00	0.49, 0.63	0.31, 0.57	Only sampled once		

Table S2: Effects of soil management on soil bacterial diversity. Table includes coefficients (β), test statistics (Z), and P-values from model averaging as well as test statistics (χ^2) and P-values from likelihood-ratio tests, comparing models with and without each predictor. Models with significant interactions always retained constituent fixed effects. Phylogenetic diversity and observed OTUs were transformed (squared) to better meet normality assumptions.

	**Shannon Diversity (no filter)**			**Shannon Diversity (0.01% filter)**			**Shannon Diversity (0.1% filter)**		
	β	Model Averaged Z, P	Model Selection χ^2, P	β	Model Averaged Z, P	Model Selection χ^2, P	β	Model Averaged Z, P	Model Selection χ^2, P
Compost	0.00	0.03, 0.98	0.23, 0.63	0.02	0.47, 0.64	1.76, 0.18	0.02	0.48, 0.63	1.55, 0.21
Cover crop	0.23	3.37, <0.01	Included	0.23	4.14, <0.01	Included	0.23	4.19, <0.01	Included
Elapsed days	0.01	2.98, <0.01	Included	0.01	4.45, <0.01	Included	0.01	4.34, <0.01	Included
Compost : Cover crop	0.04	0.40, 0.69	0.25, 0.62	0.08	1.29, 0.20	2.40, 0.12	0.07	1.23, 0.22	2.19, 0.14
Compost : Elapsed days	0.01	0.97, 0.33	1.15, 0.28	0.00	0.31, 0.75	0.12, 0.73	0.00	0.42, 0.67	0.22, 0.64
Cover crop : Elapsed days	-0.02	3.80, <0.01	13.96, <0.01	-0.02	3.82, <0.01	14.27, <0.01	-0.02	3.77, <0.01	13.92, <0.01
	**Simpson Diversity (no filter)**			**Simpson Diversity (0.01% filter)**			**Simpson Diversity (0.1% filter)**		
	β	Model Averaged Z, P	Model Selection χ^2, P	β	Model Averaged Z, P	Model Selection χ^2, P	β	Model Averaged Z, P	Model Selection χ^2, P
Compost	8.40	0.41, 0.68	Included	3.20	0.34, 0.74	Included	3.02	0.35, 0.73	Included
Cover crop	83.10	3.60, <0.01	Included	37.24	3.46, <0.01	Included	33.30	3.45, <0.01	Included
Elapsed days	3.95	2.86, <0.01	Included	2.06	3.26, <0.01	Included	1.83	3.22, <0.01	Included
Compost : Cover crop	64.62	3,00, <0.01	10.09, <0.01	29.53	2.99, <0.01	9.39, <0.01	25.76	2.93, <0.01	9.15, <0.01
Compost : Elapsed days	-2.01	1.19, 0.23	1.77, 0.18	-0.95	1.25, 0.21	1.93, 0.16	-0.87	1.28, 0.20	2.02, 0.16
Cover crop : Elapsed days	-6.92	4.10, <0.01	17.16, <0.01	-2.81	3.69, <0.01	14.18, <0.01	-2.48	3.63, <0.01	13.82, <0.01
	**Phylogenetic Diversity^2 (no filter)**			**Phylogenetic Diversity^2 (0.01% filter)**			**Phylogenetic Diversity^2 (0.1% filter)**		
	β	Model Averaged Z, P	Model Selection χ^2, P	β	Model Averaged Z, P	Model Selection χ^2, P	β	Model Averaged Z, P	Model Selection χ^2, P
Compost	-429.93	1.62, 0.11	Included	-3.43	1.98, 0.05	Included	-1.61	1.90, 0.06	Included
Cover crop	193.41	0.93, 0.35	0.18, 0.67	0.96	0.71, 0.48	0.15, 0.70	0.35	0.60, 0.55	0.26, 0.61
Elapsed days	-15.67	1.22, 0.22	Included	0.00	0.01, 0.99	Included	0.01	0.21, 0.83	Included
Compost : Cover crop	-424.86	1.70, 0.09	3.83, 0.06	-2.18	1.29, 0.20	2.41, 0.12	-0.73	0.88, 0.38	1.27, 0.26
Compost : Elapsed days	38.11	2.49, 0.01	6.73, 0.01	0.30	2.49, 0.01	6.72, 0.01	0.14	2.17, 0.03	5.20, 0.02
Cover crop : Elapsed days	-11.26	0.72, 0.47	0.65, 0.42	-0.10	0.84, 0.40	0.90, 0.34	-0.04	0.69, 0.49	0.60, 0.44
	**Observed OTUs^2 (no filter)**			**Observed OTUs^2 (0.01% filter)**			**Observed OTUs^2 (0.1% filter)**		
	β	Model Averaged Z, P	Model Selection χ^2, P	β	Model Averaged Z, P	Model Selection χ^2, P	β	Model Averaged Z, P	Model Selection χ^2, P
Compost	-250.16	1.67, 0.1	Included	-6.46	2.02, 0.04	Included	-2.94	1.87, 0.06	Included
Cover crop	114.44	0.99, 0.32	Included	1.94	0.77, 0.44	0.23, 0.63	0.62	0.53, 0.60	0.05, 0.82
Elapsed days	-10.88	1.59, 0.11	Included	0.00	0.00, 1.00	Included	0.02	0.27, 0.79	Included
Compost : Cover crop	-253.05	1.84, 0.07	4.40, 0.04	-4.14	1.33, 0.18	2.52, 0.11	-1.89	1.20, 0.23	2.10, 0.15
Compost : Elapsed days	21.79	2.62, 0.01	7.41, 0.01	0.56	2.54, 0.01	6.96, 0.01	0.24	2.21, 0.03	5.37, 0.02
Cover crop : Elapsed days	-4.78	0.56, 0.58	0.40, 0.53	-0.19	0.83, 0.41	0.87, 0.35	-0.06	0.55, 0.58	0.39, 0.53

Table S3: Effects of soil management on bacterial community composition. Table includes test statistics (F values; first number) and P-values (second number) from PERMANOVA. First two rows constitute core analyses, whereas the next 6 rows correspond to post-hoc analyses comparing pairs of soil treatments.

	Bray-curtis (no filter)	Bray-curtis (0.01% filter)	Bray-curtis (0.1% filter)	Euclidian (no filter)	Euclidian (0.01% filter)	Euclidian (0.1% filter)	Unifrac (no filter)	Unifrac (0.01% filter)	Unifrac (0.1% filter)
Elapsed days	3.18, <0.01	3.85, <0.01	4.53, <0.01	6.81, <0.01	3.42, <0.01	10.32, <0.01	6.94, <0.01	3.25, <0.01	11.09, <0.01
Overall treatment	8.31, <0.01	9.17, <0.01	11.28, <0.01	2.86, <0.01	8.04, <0.01	4.11, <0.01	3.11, <0.01	7.72, <0.01	4.24, <0.01
Compost/cover crop vs. Compost only	16.4, <0.01	16.21, <0.01	21.17, <0.01	12.71, <0.01	13.77, <0.01	18.78, <0.01	13.13, <0.01	12.57, <0.01	19.25, <0.01
Compost/cover crop vs. Cover crop only	5.45, <0.01	5.08, <0.01	6.20, <0.01	4.43, <0.01	4.45, <0.01	5.75, <0.01	4.61, <0.01	4.64, <0.01	6.14, <0.01
Compost/cover crop vs. No compost/cover crop	6.61, <0.01	7.48, <0.01	9.18, <0.01	5.55, <0.01	6.45, <0.01	8.31, 0.01	5.69, <0.01	5.89, <0.01	10.55, 0.01
Compost only vs. Cover crop only	9.12, <0.01	10.30, <0.01	12.64, <0.01	7.44, <0.01	9.31, <0.01	11.89, <0.01	7.36, <0.01	8.93, <0.01	11.24, <0.01
Compost only vs. No compost/cover crop	8.20, <0.01	8.81, <0.01	10.52, <0.01	6.76, <0.01	7.99, <0.01	9.95, <0.01	7.02, <0.01	7.83, <0.01	9.53, <0.01
Cover crop only vs. No compost/cover crop	9.13, <0.01	10.31, <0.01	12.64, <0.01	7.44, <0.01	9.31, <0.01	11.89, <0.01	7.36, <0.01	8.93, <0.01	11.24, <0.01

Table S4: Changes in the dissimilarity between bacterial communities present in compost versus non-compost treatments over the growing season (measured as multivariate distances from each 'compost sample' to the centroid of all non-compost samples; see methods). Table includes coefficients (β), test statistics (Z), and P-values from model averaging as well as test statistics (χ^2) and P-values from likelihood-ratio tests, comparing models with and without each predictor.

	β	Model Averaged Z, P	Model Selection χ^2, P
Bray-curtis (no filter)	-0.01	3.52, <0.01	11.25, <0.01
Bray-curtis (0.01% filter)	-0.01	1.99, 0.05	4.42, 0.04
Bray-curtis (0.1% filter)	-0.01	1.67, 0.10	3.20, 0.07
Euclidian (no filter)	-0.01	3.05, <0.01	9.03, <0.01
Euclidian (0.01% filter)	-0.01	2.06, 0.04	4.71, 0.03
Euclidian (0.1% filter)	-0.01	1.76, 0.08	3.49, 0.06
Unifrac (no filter)	-0.01	2.79, 0.01	7.86, 0.01
Unifrac (0.01% filter)	-0.02	1.95, 0.05	4.24, 0.04
Unifrac (0.1% filter)	-0.01	1.52, 0.13	2.70, 0.10

Table S5: Effects of soil physicochemical properties on soil bacterial diversity. Table includes coefficients (β), test statistics (Z), and P-values from model averaging as well as test statistics (χ^2) and P-values from likelihood-ratio tests, comparing models with and without each predictor. Phylogenetic diversity and observed OTUs were transformed (squared) to better meet normality assumptions. As a result of high collinearity, two analyses per diversity metric were conducted: one for macronutrients, micronutrients, physical properties, and moisture content and another for pH and organic matter.

	**Shannon Diversity (no filter)**			**Shannon Diversity (0.01% filter)**			**Shannon Diversity (0.1% filter)**		
	β	Model Averaged Z, P	Model Selection χ^2, P	β	Model Averaged Z, P	Model Selection χ^2, P	β	Model Averaged Z, P	Model Selection χ^2, P
Macronutrients (PC 1)	0.00	1.5, 0.13	3.50, 0.06	0.00	1.41, 0.16	1.51, 0.22	0.00	1.57, 0.12	1.91, 0.17
Micronutrients (PC 1)	-0.02	2.53, 0.01	7.94, <0.01	-0.02	3.51, <0.01	11.15, <0.01	-0.02	3.51, <0.01	11.10, <0.01
Moisture content	0.02	1.34, 0.18	1.14, 0.29	0.03	2.38, 0.02	6.45, 0.01	0.03	2.26, 0.02	6.30, 0.01
Physical properties (PC 1)	0.00	0.26, 0.79	0.07, 0.78	-0.01	1.57, 0.12	3.62, 0.06	-0.01	1.53, 0.13	3.53, 0.06
pH	0.10	0.76, 0.44	1.17, 0.28	0.11	1.1, 0.27	1.26, 0.26	0.12	1.22, 0.22	1.51, 0.22
Organic Matter	0.11	1.73, 0.08	4.15, 0.04	0.16	3.39, <0.01	10.54, <0.01	0.17	3.45, <0.01	11.11, <0.01
	**Simpson Diversity (no filter)**			**Simpson Diversity (0.01% filter)**			**Simpson Diversity (0.1% filter)**		
	β	Model Averaged Z, P	Model Selection χ^2, P	β	Model Averaged Z, P	Model Selection χ^2, P	β	Model Averaged Z, P	Model Selection χ^2, P
Macronutrients (PC 1)	0.30	3.60, <0.01	13.35, <0.01	0.11	3.04, <0.01	9.94, <0.01	0.10	3.07, <0.01	10.15, <0.01
Micronutrients (PC 1)	-8.48	3.42, <0.01	14.61, <0.01	-4.52	4.12, <0.01	18.6, <0.01	-4.03	4.14, <0.01	18.67, <0.01
Moisture content	11.61	2.13, 0.03	5.55, 0.02	6.22	2.55, 0.01	7.51, <0.01	5.44	2.52, 0.01	7.30, 0.01
Physical properties (PC 1)	-2.48	1.71, 0.09	3.98, 0.05	-1.64	2.50, 0.01	7.29, <0.01	-1.46	2.52, 0.01	7.40, 0.01
pH	55.62	1.43, 0.15	2.23, 0.14	25.74	1.47, 0.14	2.28, 0.13	23.01	1.48, 0.14	2.32, 0.13
Organic Matter	107.23	5.09, <0.01	21.22, <0.01	49.72	5.29, <0.01	19.74, <0.01	44.39	5.35, <0.01	20.10, <0.01
	**Observed OTUs^2 (no filter)**			**Observed OTUs^2 (0.01% filter)**			**Observed OTUs^2 (0.1% filter)**		
	β	Model Averaged Z, P	Model Selection χ^2, P	β	Model Averaged Z, P	Model Selection χ^2, P	β	Model Averaged Z, P	Model Selection χ^2, P
Macronutrients (PC 1)	-0.14	0.35, 0.73	0.01, 0.92	-0.01	0.74, 0.46	0.16, 0.69	0.00	0.73, 0.47	0.12, 0.73
Micronutrients (PC 1)	0.42	0.03, 0.97	0.12, 0.73	-0.21	0.68, 0.5	0.49, 0.48	-0.10	0.65, 0.52	0.44, 0.51
Moisture content	-22.42	0.83, 0.41	0.85, 0.36	-0.71	1.05, 0.29	1.25, 0.26	-0.46	1.37, 0.17	2.08, 0.15
Physical properties (PC 1)	9.32	1.05, 0.30	1.50, 0.22	0.06	0.33, 0.74	0.04, 0.85	0.03	0.32, 0.75	0.04, 0.84
pH	18.53	0.09, 0.93	0.01, 0.91	-0.29	0.06, 0.95	0.06, 0.81	0.49	0.21, 0.83	0.00, 0.99
Organic Matter	-72.85	0.65, 0.52	0.43, 0.51	-1.07	0.43, 0.67	0.25, 0.62	-0.74	0.59, 0.55	0.33, 0.57
	**Phylogenetic Diversity^2 (no filter)**			**Phylogenetic Diversity^2 (0.01% filter)**			**Phylogenetic Diversity^2 (0.1% filter)**		
	β	Model Averaged Z, P	Model Selection χ^2, P	β	Model Averaged Z, P	Model Selection χ^2, P	β	Model Averaged Z, P	Model Selection χ^2, P
Macronutrients (PC 1)	-0.24	0.32, 0.75	0.00, 1.00	0.00	0.75, 0.45	0.17, 0.68	0.00	0.57, 0.57	0.02, 0.89
Micronutrients (PC 1)	-3.03	0.13, 0.89	0.03, 0.86	-0.10	0.62, 0.54	0.41, 0.52	-0.06	0.71, 0.48	0.61, 0.43
Moisture content	-47.99	0.98, 0.33	1.17, 0.28	-0.39	1.06, 0.29	1.27, 0.26	-0.24	1.37, 0.17	2.09, 0.15
Physical properties (PC 1)	15.74	0.99, 0.32	1.37, 0.24	0.04	0.35, 0.72	0.05, 0.82	0.02	0.5, 0.62	0.18, 0.67
pH	44.67	0.12, 0.90	0.01, 9.94	-0.30	0.12, 0.9	0.10, 0.76	-0.30	0.12, 0.9	0.00, 0.95
Organic Matter	-122.84	0.61, 0.54	0.37, 0.54	-0.62	0.46, 0.64	0.31, 0.58	-0.62	0.46, 0.64	0.18, 0.67

Table S6: Effects of soil physicochemical properties on bacterial community composition. Table includes test statistics (F values; first number) and P-values (second number) from PERMANOVA. As a result of high collinearity, two analyses per community composition metric were conducted: one for macronutrients, micronutrients, physical properties, and moisture content and another for pH and organic matter.

	**Macronutrients (PC 1)**	**Micronutrients (PC 1)**	**Physical properties (PC 1)**	**Moisture content**	**pH**	**Organic matter**
Bray-curtis (no filter)	6.78, <0.01	4.56, 0.01	1.99, <0.01	1.35, 0.61	4.01, <0.01	8.46, <0.01
Bray-curtis (0.01% filter)	6.20, <0.01	5.30, 0.03	2.24, <0.01	1.54, 0.38	4.68, <0.01	8.56, 0.06
Bray-curtis (0.1% filter)	7.56, <0.01	6.10, 0.01	2.54, <0.01	1.67, 0.27	5.47, <0.01	10.22, 0.03
Euclidian (no filter)	5.75, <0.01	3.97, 0.01	1.84, <0.01	1.34, 0.51	3.45, <0.01	7.16, <0.01
Euclidian (0.01% filter)	5.71, <0.01	4.76, 0.03	2.29, <0.01	1.48, 0.42	4.25, <0.01	7.62, 0.03
Euclidian (0.1% filter)	7.18, <0.01	5.75, 0.01	2.73, <0.01	1.64, 0.33	5.11, 0.01	9.42, 0.02
Unifrac (no filter)	5.78, <0.01	4.18, 0.01	2.07, <0.01	1.28, 0.64	3.77, <0.01	7.23, <0.01
Unifrac (0.01% filter)	5.57, <0.01	4.33, 0.21	2.32, <0.01	1.37, 0.56	3.90, 0.02	7.38, 0.04
Unifrac (0.1% filter)	7.63, <0.01	5.98, 0.01	2.27, <0.01	2.23, 0.05	6.09, 0.02	10.23, 0.36

Table S7: Effects of soil physicochemical properties on pathogen suppression (fraction of *Salmonella* or *Listeria* remaining after 10- and 30-day incubations). *Salmonella* and *Listeria* suppression rates were log and quarter-root transformed to meet normality assumptions (see methods). Table includes coefficients (β), test statistics (Z), and P-values from model averaging as well as test statistics (χ^2) and P-values from likelihood-ratio tests, comparing models with and without each predictor. As a result of high collinearity, two analyses per community composition metric were conducted: one for macronutrients, micronutrients, physical properties, and moisture content and another for pH and organic matter.

	**Salmonella (10 day incubation)**			**Salmonella (30 day incubation)**			**Listeria (10 day incubation)**			**Listeria (30 day incubation)**		
	β	Model Averaged Z, P	Model Selection χ^2, P	β	Model Averaged Z, P	Model Selection χ^2, P	β	Model Averaged Z, P	Model Selection χ^2, P	β	Model Averaged Z, P	Model Selection χ^2, P
Macronutrients (PC 1)	-0.01	3.07, <0.01	10.2, <0.01	-0.02	3.59, <0.01	13.0, <0.01	0.00	6.31, <0.01	36.5, <0.01	0.00	0.73, 0.46	0.16, 0.69
Micronutrients (PC 1)	-0.20	2.13, 0.03	5.01, 0.03	-0.34	2.24, 0.03	5.06, 0.02	-0.01	1.60, 0.11	3.96, 0.05	0.00	1.47, 0.14	1.75, 0.19
Moisture content	0.31	1.31, 0.19	2.05, 0.15	0.67	1.90, 0.06	3.87, 0.05	-0.01	0.45, 0.65	0.15, 0.69	-0.01	0.88, 0.38	0.84, 0.36
Physical properties (PC 1)	-0.01	0.15, 0.88	0.27, 0.60	-0.09	1.06, 0.29	2.20, 0.14	-0.01	1.62, 0.11	4.78, 0.03	0.00	0.36, 0.72	0.60, 0.44
pH	1.07	0.65, 0.52	0.09, 0.76	2.70	1.06, 0.29	0.53, 0.47	0.13	1.10, 0.27	1.18, 0.28	-0.03	0.68, 0.50	0.86, 0.35
Organic Matter	-1.25	1.60, 0.12	1.91, 0.17	-1.97	1.53, 0.13	1.55, 0.22	-0.17	2.91, <0.01	6.41, 0.01	-0.02	1.02, 0.31	1.68, 0.20

Table S8: Effects of soil bacterial diversity and community composition on pathogen suppression (fraction of *Salmonella* or *Listeria* remaining after 10- and 30-day incubations). *Salmonella* and *Listeria* suppression rates were log and quarter-root transformed to meet normality assumptions (see methods). Table includes coefficients (β), test statistics (Z), and P-values from model averaging as well as test statistics (χ^2) and P-values from likelihood-ratio tests, comparing models with and without each predictor. Separate analyses were conducted for each diversity metrics and each measure of community composition.

	**Salmonella (10 day incubation)**			**Salmonella (30 day incubation)**			**Listeria (10 day incubation)**			**Listeria (30 day incubation)**		
	β	Model Averaged Z, P	Model Selection χ^2, P	β	Model Averaged Z, P	Model Selection χ^2, P	β	Model Averaged Z, P	Model Selection χ^2, P	β	Model Averaged Z, P	Model Selection χ^2, P
Shannon Diversity (no filter)	0.46	0.75, 0.46	0.62, 0.43	0.69	0.40, 0.69	0.18, 0.67	-0.11	0.71, 0.48	0.50, 0.48	-0.06	0.99, 0.32	1.05, 0.31
Shannon Diversity (0.01% filter)	0.17	1.38, 0.17	2.08, 0.15	0.19	1.31, 0.19	1.89, 0.17	-0.09	0.50, 0.62	0.22, 0.64	-0.14	2.19, 0.03	5.10, 0.02
Shannon Diversity (0.1% filter)	0.21	1.25, 0.21	1.71, 0.19	0.24	1.16, 0.24	1.49, 0.22	-0.11	0.62, 0.53	0.37, 0.54	-0.14	2.17, 0.03	4.97, 0.03
Simpson Diversity (no filter)	0.92	0.10, 0.92	0.01, 0.92	0.65	0.46, 0.65	0.24, 0.63	0.00	2.19, 0.03	5.02, 0.02	0.00	1.95, 0.05	4.05, 0.04
Simpson Diversity (0.01% filter)	0.73	0.34, 0.73	0.13, 0.72	0.85	0.19, 0.85	0.04, 0.84	0.00	1.55, 0.12	2.57, 0.11	0.00	2.10, 0.04	4.71, 0.03
Simpson Diversity (0.1% filter)	0.76	0.31, 0.76	0.10, 0.75	0.87	0.17, 0.87	0.03, 0.86	0.00	1.57, 0.12	2.62, 0.11	0.00	2.10, 0.04	4.68, 0.03
# OTUs (no filter)	0.93	0.09, 0.93	0.01, 0.93	0.99	0.01, 0.99	0.00, 0.99	0.00	0.65, 0.52	0.46, 0.50	0.00	1.92, 0.06	3.91, 0.05
# OTUs (0.01% filter)	0.16	1.41, 0.16	2.18, 0.14	0.14	1.47, 0.14	2.36, 0.12	0.00	1.47, 0.14	2.33, 0.13	0.00	0.92, 0.36	0.94, 0.33
# OTUs (0.1% filter)	0.20	1.28, 0.20	1.79, 0.18	0.16	1.40, 0.16	2.15, 0.14	0.00	1.49, 0.14	2.42, 0.12	0.00	0.99, 0.32	1.09, 0.30
PD (no filter)	0.97	0.03, 0.97	0.00, 0.97	0.92	0.10, 0.92	0.01, 0.92	0.00	0.70, 0.48	0.55, 0.46	0.00	1.81, 0.07	3.51, 0.06
PD (0.01% filter)	0.16	1.42, 0.16	2.21, 0.14	0.13	1.51, 0.13	2.49, 0.11	0.00	1.50, 0.13	2.42, 0.12	0.00	1.01, 0.31	1.13, 0.29
PD (0.1% filter)	0.17	1.37, 0.17	2.05, 0.15	0.15	1.45, 0.15	2.30, 0.13	0.00	1.52, 0.13	2.52, 0.11	0.00	1.13, 0.26	1.40, 0.24
Bray-Curtis Axis 1 (no filter)	0.82	0.03, 0.98	0.00, 0.98	42.40	0.42, 0.67	0.20, 0.65	0.06	1.90, 0.06	3.90, 0.05	1.00	0.00, 1.00	0.01, 0.91
Bray-Curtis Axis 2 (no filter)	202.68	4.03, <0.01	15.50, <0.01	804.85	9.37, <0.01	26.05, <0.01	0.00	5.21, <0.01	22.56, <0.01	0.22	1.23, 0.22	0.53, 0.47
Bray-Curtis Axis 1 (0.01% filter)	-5.85	0.96, 0.34	1.05, 0.31	-11.01	1.21, 0.23	1.65, 0.20	0.37	0.90, 0.37	0.89, 0.35	0.94	0.08, 0.94	0.01, 0.94
Bray-Curtis Axis 2 (0.01% filter)	34.12	3.72, <0.01	13.69, <0.01	61.51	4.46, <0.01	18.77, <0.01	0.00	5.15, <0.01	23.28, <0.01	0.99	0.01, 0.99	0.00, 1.00
Bray-Curtis Axis 1 (0.1% filter)	5.86	0.70, 0.49	0.55, 0.46	12.35	0.99, 0.32	1.11, 0.29	0.26	1.13, 0.26	1.38, 0.24	0.96	0.05, 0.96	0.01, 0.92
Bray-Curtis Axis 2 (0.1% filter)	49.44	3.85, <0.01	14.42, <0.01	90.61	4.75, <0.01	20.66, <0.01	0.00	5.08, <0.01	22.16, <0.01	0.44	0.77, 0.44	0.34, 0.56
Euclidian Axis 1 (no filter)	0.00	0.10, 0.92	0.01, 0.91	0.01	0.12, 0.91	0.02, 0.90	0.06	1.87, 0.06	3.74, 0.05	0.99	0.01, 0.99	0.01, 0.92
Euclidian Axis 2 (no filter)	0.13	4.28, <0.01	17.13, <0.01	0.48	9.96, <0.01	31.22, <0.01	0.00	5.63, <0.01	25.84, <0.01	0.19	1.31, 0.19	0.61, 0.44
Euclidian Axis 1 (0.01% filter)	-0.02	0.82, 0.41	0.76, 0.38	-0.03	1.05, 0.29	1.25, 0.26	0.28	1.08, 0.28	1.27, 0.26	0.95	0.06, 0.95	0.01, 0.94
Euclidian Axis 2 (0.01% filter)	0.11	3.74, <0.01	13.76, <0.01	0.20	4.49, <0.01	18.89, <0.01	0.00	5.13, <0.01	23.20, <0.01	0.86	0.18, 0.86	0.02, 0.89
Euclidian Axis 1 (0.1% filter)	-0.02	0.66, 0.51	0.49, 0.48	-0.04	0.93, 0.35	0.98, 0.32	0.23	1.19, 0.23	1.52, 0.22	0.96	0.05, 0.96	0.01, 0.93
Euclidian Axis 2 (0.1% filter)	0.15	3.81, <0.01	14.14, <0.01	0.28	4.66, <0.01	20.00, <0.01	0.00	5.04, <0.01	21.92, <0.01	0.61	0.52, 0.61	0.15, 0.70
Unifrac Axis 1 (no filter)	-40.03	0.32, 0.75	0.12, 0.73	-38.97	0.12, 0.90	0.02, 0.90	0.06	1.91, 0.06	3.91, 0.05	0.95	0.06, 0.95	0.02, 0.88
Unifrac Axis 2 (no filter)	755.29	3.99, <0.01	15.26, <0.01	2599.18	8.59, <0.01	27.27, <0.01	0.00	5.95, <0.01	29.2, <0.01	0.34	0.95, 0.34	0.27, 0.60
Unifrac Axis 1 (0.01% filter)	-8.95	0.38, 0.71	0.16, 0.69	-16.27	0.45, 0.65	0.24, 0.63	0.06	1.86, 0.06	3.81, 0.05	0.99	0.01, 0.99	0.00, 1.00
Unifrac Axis 2 (0.01% filter)	136.91	4.19, <0.01	16.59, <0.01	241.66	4.92, <0.01	21.65, <0.01	0.00	5.05, <0.01	23.18, <0.01	0.66	0.44, 0.66	0.19, 0.66
Unifrac Axis 1 (0.1% filter)	-14.17	0.50, 0.61	0.29, 0.59	-31.35	0.73, 0.47	0.61, 0.44	0.23	1.19, 0.23	1.47, 0.23	0.93	0.08, 0.93	0.01, 0.93
Unifrac Axis 2 (0.1% filter)	140.87	3.38, <0.01	11.42, <0.01	246.73	3.85, <0.01	14.45, <0.01	0.00	4.18, <0.01	15.45, <0.01	0.90	0.12, 0.90	0.01, 0.91

Table S9: Effects of soil management on pathogen suppression (fraction of *Salmonella* or *Listeria* remaining after 10- and 30-day incubations). *Salmonella* and *Listeria* suppression rates were log and quarter-root transformed to meet normality assumptions (see methods). Table includes coefficients (β), test statistics (Z), and P-values from model averaging as well as test statistics (χ^2) and P-values from likelihood-ratio tests, comparing models with and without each predictor.

	**Salmonella (10 day incubation)**			**Salmonella (30 day incubation)**			**Listeria (10 day incubation)**			**Listeria (30 day incubation)**		
	β	Model Averaged Z, P	Model Selection χ^2, P	β	Model Averaged Z, P	Model Selection χ^2, P	β	Model Averaged Z, P	Model Selection χ^2, P	β	Model Averaged Z, P	Model Selection χ^2, P
Compost	-1.32	2.41, 0.02	Included	-1.44	2.60, 0.01	Included	-0.01	1.94, 0.05	7.19, 0.01	-0.11	5.21, <0.01	Included
Cover crop	-0.25	0.54, 0.59	1.23, 0.27	-0.47	1.01, 0.31	0.51, 0.48	-0.07	1.69, 0.09	5.44, 0.02	-0.01	0.57, 0.57	0.39, 0.53
Elapsed days	2.27	8.97, <0.01	Included	3.14	13.6, <0.01	Included	0.03	2.75, 0.01	25.99, <0.01	-0.01	1.88, 0.06	Included
Elapsed days^2	-0.34	7.90, <0.01	Included	-0.42	10.5, <0.01	Included	0.00	0.22, 0.83	15.96, <0.01	0.00	0.57, 0.57	2.65, 0.10
Elapsed days^3	0.01	7.49, <0.01	41.0, <0.01	0.02	9.19, <0.01	52.5, <0.01	0.00	0.92, 0.36	0.06, 0.81	0.00	0.20, 0.84	0.84, 0.36
Compost : Cover crop	0.6	1.03, 0.30	1.64, 0.20	0.85	1.57, 0.12	3.47, 0.06	-0.02	0.29, 0.78	0.13, 0.72	0.01	0.53, 0.60	0.46, 0.50
Compost : Elapsed days	0.11	2.67, 0.01	7.98, <0.01	0.08	2.24, 0.02	5.82, 0.02	0.01	1.24, 0.21	1.87, 0.17	0.01	4.25, 0.01	17.4, <0.01
Cover crop : Elapsed days	-0.05	1.13, 0.26	1.67, 0.20	0.03	0.86, 0.39	0.98, 0.32	0.00	0.49, 0.63	0.31, 0.58	0.00	0.59, 0.56	0.45, 0.50

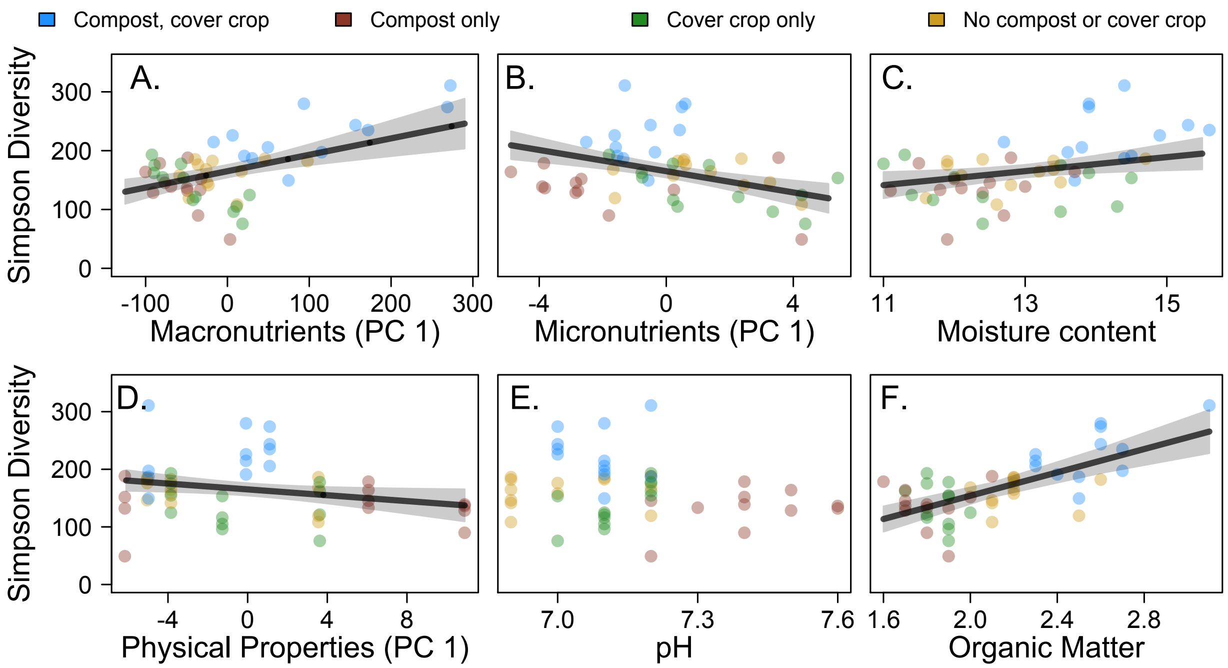

Fig. S1: Effects of soil physicochemical properties on soil bacterial diversity. Increases in macronutrient concentrations (principal component axis 1; Panel A), moisture content (Panel C), and organic matter (Panel F) are associated with higher levels of bacterial diversity (Simpson metric). In contrast, Simpson diversity declines in soils with lower micronutrient concentrations (principal component axis 1; Panel B) and sandier soils (physical properties principal component axis 1; Panel D). Points represent soil samples, colored by management treatment. Lines represent predictions from linear mixed models (LMM); shaded regions are 95% confidence intervals.

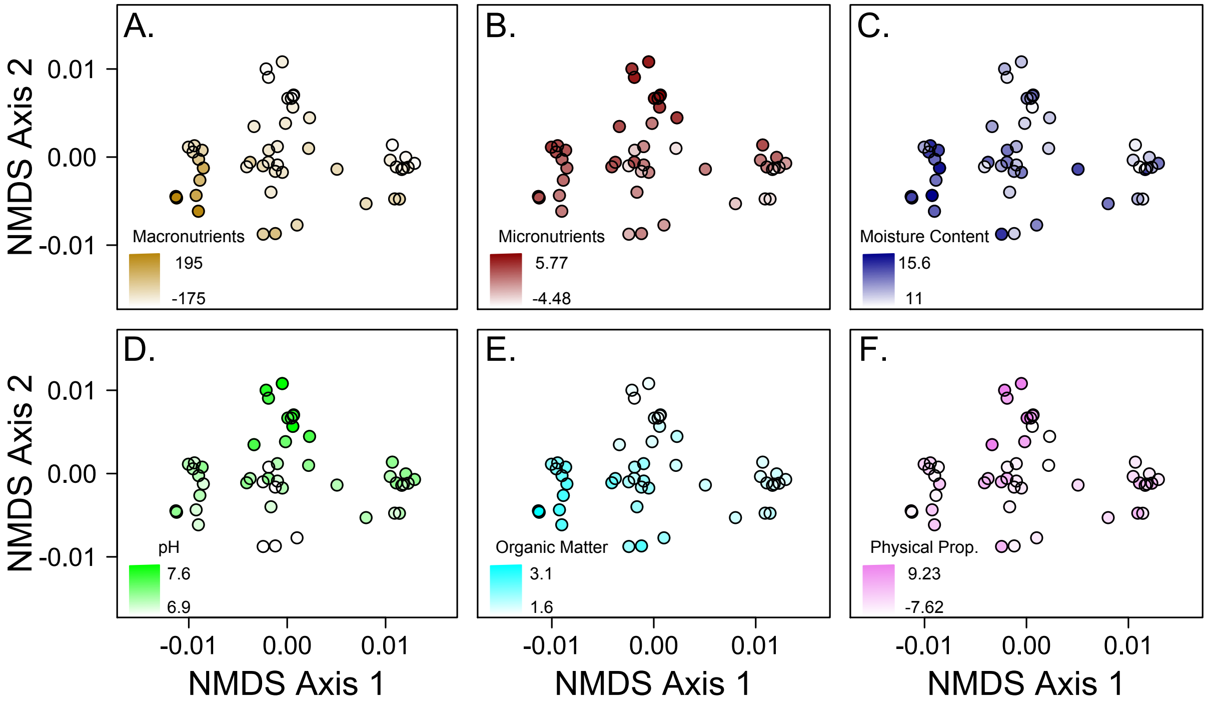

Fig. S2: Effects of soil physicochemical properties on bacterial community composition. Bacterial communities shifted among soil samples with different levels of macronutrients (principal component axis 1; Panel A), micronutrients (principal component axis 1; Panel B), pH (Panel D), organic matter (Panel E), and physical properties (principal component axis 1; Panel F). Soil moisture (Panel C) content did not affect community composition. Points represent soil samples, colored according to their physicochemical properties. Distances between points correspond to differences in bacterial community composition under non-metric multidimensional scaling (NMDS).

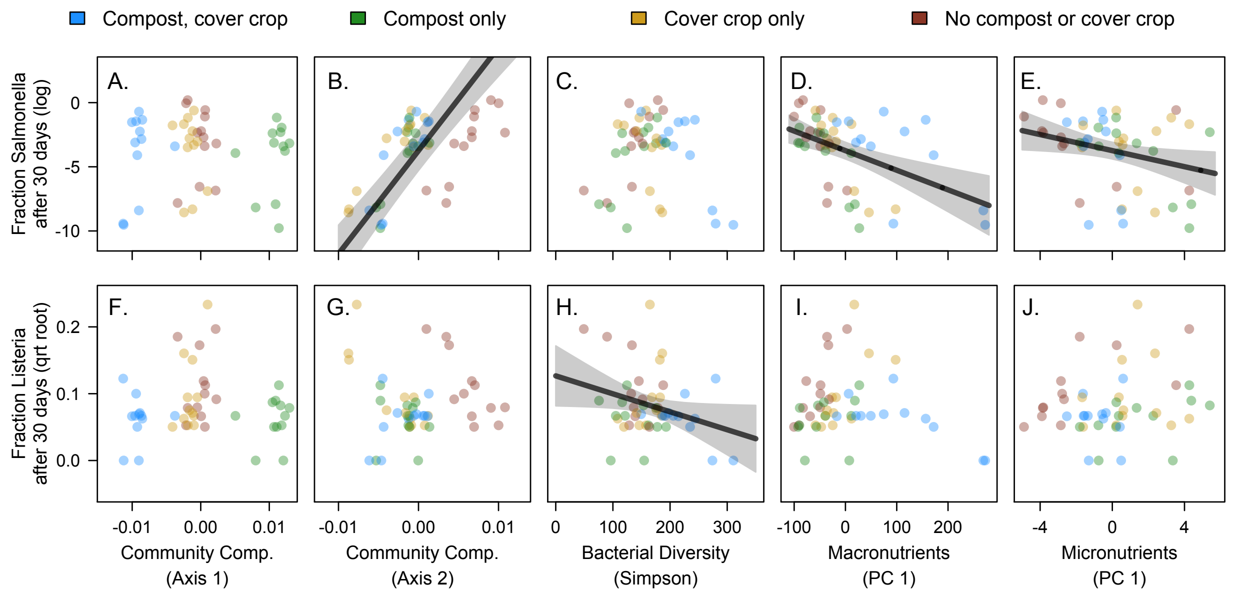

Fig. S3: Effects of soil physicochemical properties and bacterial communities on pathogen suppression. Pathogen suppression is measured as the Mean Probable Number (MPN) of *Salmonella* or *Listeria* after 30 days of incubation divided by the initial MPN of the inoculant (followed by log and quarter-root transformations for *Salmonella* and *Listeria*, respectively; see methods). *Salmonella* concentrations exhibited steeper declines in soils with more macronutrients (Panel D), more micronutrients (Panel E), and bacterial communities (Panel B) more similar to those present in conventional soil treatments (*i.e.,* no compost or cover crop; NMDS axis 2 in Fig. 2b). The first NMDS axis explaining variation in bacterial community composition (Panel A) and bacterial diversity (Simpson index; Panel C) did not affect *Salmonella* suppression. In contrast, *Listeria* exhibited steeper reductions in soils with more bacterial diversity (Panel H) but was not affected by bacterial community composition (Panels F/G), macronutrients (Panel I), or micronutrients (Panel J). Points represent soil samples, colored by management treatment. Lines represent predictions from linear mixed models (LMM); shaded regions are 95% confidence intervals.

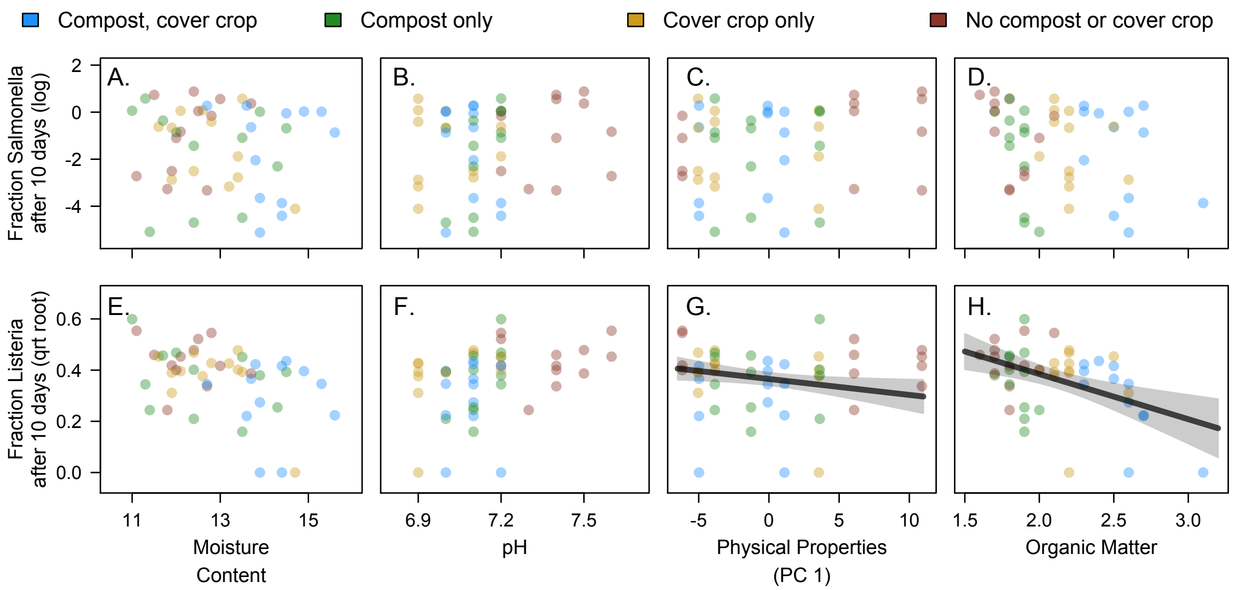

Fig. S4: Effects of soil physicochemical properties on pathogen suppression after 10 days of incubation. *Salmonella* concentration reductions after 10 days of incubation in soils were not influenced by soil moisture content (Panel A), pH (Panel B), physical properties (Panel C), or organic matter (Panel D). Moisture content (Panel E) and pH (Panel F) also did not influence *Listeria* reductions; however, less *Listeria* persisted after 10 days of incubation in sandier soils (Panel G; physical properties principal components axis 1) and soils with more organic matter (Panel H). Points represent soil samples, colored by management treatment. Lines represent predictions from linear mixed models (LMM); shaded regions are 95% confidence intervals.

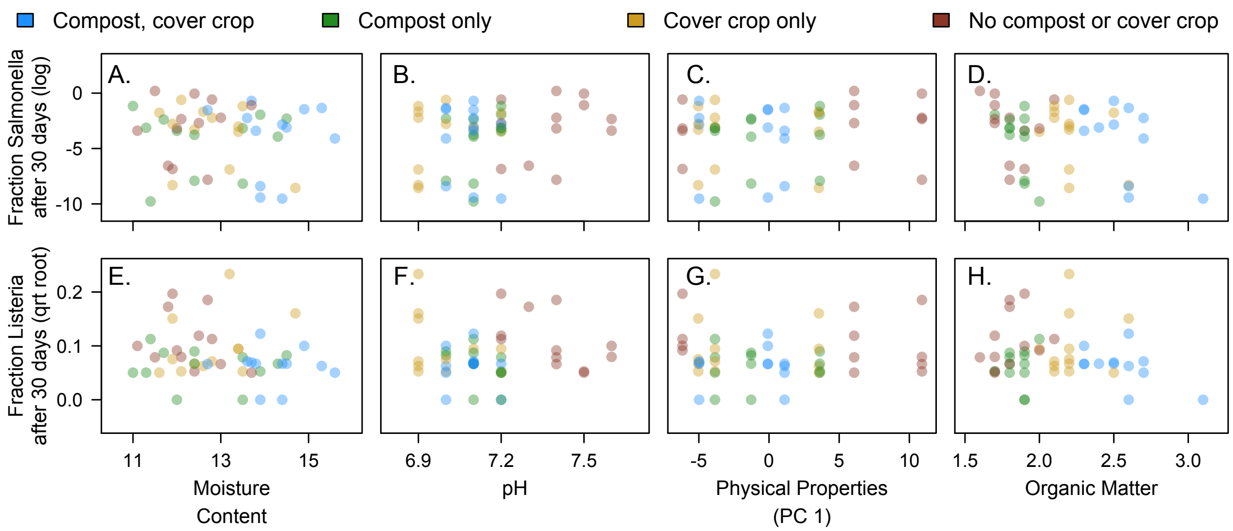

Fig. S5: Effects of soil physicochemical properties on pathogen suppression after 30 days of incubation. *Salmonella* and *Listeria* concentration reductions after 30 days of incubation in soils were not influenced by soil moisture content (Panel A/E), pH (Panel B/F), physical properties (Panel C/G), or organic matter (Panel D/H). Points represent soil samples, colored by management treatment. Lines represent predictions from linear mixed models (LMM); shaded regions are 95% confidence intervals.
